# Higher risk of revision in total knee arthroplasty after high tibial osteotomy: a systematic review and updated meta-analysis

**DOI:** 10.1186/s12891-020-3177-9

**Published:** 2020-03-06

**Authors:** Xi Chen, Zhen Yang, Hairui Li, Shibai Zhu, Yiou Wang, Wenwei Qian

**Affiliations:** 1 Department of Orthopedic Surgery, Peking Union Medical College Hospital, Peking Union Medical College, Chinese Academy of Medical Science, Beijing, China; 2 Department of Orthopedic Surgery, Guizhou Provincial People’s Hospital, Guiyang, Guizhou China; 3 Department of Plastic Surgery, Peking Union Medical College Hospital, Peking Union Medical College, Chinese Academy of Medical Science, Beijing, China

**Keywords:** High tibial osteotomy, Total knee arthroplasty, Revision

## Abstract

**Background:**

High tibial osteotomy is commonly performed in young patients with high activity demand. Several studies have reported outcome of HTO. The reported 10-year survival ranged from 79 to 97.6%. The reported 15-year survival ranged from 56 to 65.5%, resulting in the need for conversion to TKA. Primary TKA now provides satisfactory long-term outcome in terms of function and survival. Researches have been conducted to compare clinical outcome between primary TKA and TKA after HTO to see if TKA should be the prior treatment rather than HTO in some cases. But the results were inconsistent. This study aims to compare the risk of revision and other parameters between total knee arthroplasty after high tibial osteotomy and primary total knee arthroplasty.

**Methods:**

Searches and screens of the relevant literature were conducted, after which data were extracted and pooled analysis was performed to compare the clinical outcomes between the two groups.

**Results:**

A total of 14 studies with 144,692 cases were included. Pooled analysis showed significantly more revisions and complications, and more tibial component loosening and impingement in postoperative X-ray in the HTO-TKA group. Surgical complexity during conversion to total knee arthroplasty was summarised and listed in table.

**Conclusion:**

High tibial osteotomy offers satisfactory pain relief and functional outcome in selected patients with high activity demand. However, the need for subsequent TKA should be noted, which might be a technically challenging procedure with significantly higher risk of revision comparing to primary TKA.

## Background

High tibial osteotomy (HTO) is a well-established procedure for uni-compartmental osteoarthritis of the knee and is commonly applied to young patients with high activity demands. There has been an increasing interest in high tibial osteotomy in some countries over the past decade [1]. The goal of this operation lies in correction of the mechanical axis of the lower limb, reducing the load stress of the pathological medial compartment. HTO has been reported to achieve satisfactory short-term clinical result, many of them regain satisfactory functional outcome. However, most patients who undergo this operation are relatively young, with high activity demand. Several studies have reported outcome of HTO. The reported 10-year survival ranged from 79 to 97.6%. The reported 15-year survival ranged from 56 to 65.5% [2, 3]. A meta-analysis reported 84.4% survival with 9 to 12 years follow up. It is reasonable to deduce that subsequent TKA is required in these patients [4]. These conversions to TKA are more technically demanding than primary TKA and may lead to inferior survival and functional outcomes comparing to primary TKA. Researches have been conducted to compare risk of revision and functional outcome between primary TKA and TKA after HTO, which reported controversial results. Some studies found HTO-TKA is at higher risk of revision and complication [5–7], while other studies reported similar outcome between 2 groups [8–11].

A previous meta-analysis published in 2013 reported similar outcomes between TKA following HTO and primary TKA in terms of survival and perioperative complications [12]. Most studies included in the previous meta-analysis investigated cohorts of small sample size. Several studies with larger cohorts have been published since then, some of which reported inferior survival and clinical outcomes in cases underwent TKA after HTO [5, 8, 9, 13]. Therefore, an updated meta-analysis and systematic review was conducted to compare risk of revision and other clinical parameters between TKA after HTO and primary TKA.

## Methods

### Search strategy

MEDLINE, Embase and the Cochrane Library were thoroughly searched by two independent researchers in April 2018. Search terms included tibial osteotomy, knee, replacement, arthroplasty and related MeSH terms.

### Inclusion and exclusion criteria

Studies were included if they (1) included patients undergoing TKA following HTO and patients undergoing primary TKA; (2) compared risk of revision between HTO after TKA and primary TKA (providing exact number of cases of primary TKAs, revision of primary TKAs, TKAs after HTO, Revision in TKAs after HTO); Studies were excluded if they (1) did not report quantitative data; (2) were a conference abstract, animal studies, cadaveric studies or in vitro studies.

### Data extraction and quality assessment

Data were collected and reviewed by two independent researchers. The quality of included studies was evaluated according to the Newcastle-Ottawa Scale (NOS).

### Statistical analysis

Data of interest were extracted and analysed using Review Manager 5.2 and STATA 14 as the forest plot produced by Review Manager offers more detailed information and STATA provides more options for heterogeneity assessment. All data and analysis were cross-examined. Peto’s method was utilized if incidence is considered rare; other discontinuous variables were analysed by odds ratios (ORs). Continuous data with mean and SD were analysed by weighted mean differences (WMDs). Heterogeneity among studies was assessed using the χ^2^ test, I^2^ and L’abbe test. Generally, a fixed-effects model was applied when I^2^ < 50%, and a random-effects model when I^2^ > 50%. A *p* value < 0.05 was considered statistically significant. If the analysis was conducted with Peto’s method, a fixed effect model was applied. When trials had no event in one arm or another, a small quantity (0.5) of the cell counts would be added to avoid division by zero errors as suggested in the Systematic Reviews in Health Care: Meta-Analysis in Context. It is also suggested when the count is zero in both arms, the risk difference is zero. Publication bias is evaluated by funnel plot if needed.

## Results

### Study characteristics

**Fig. 1:**
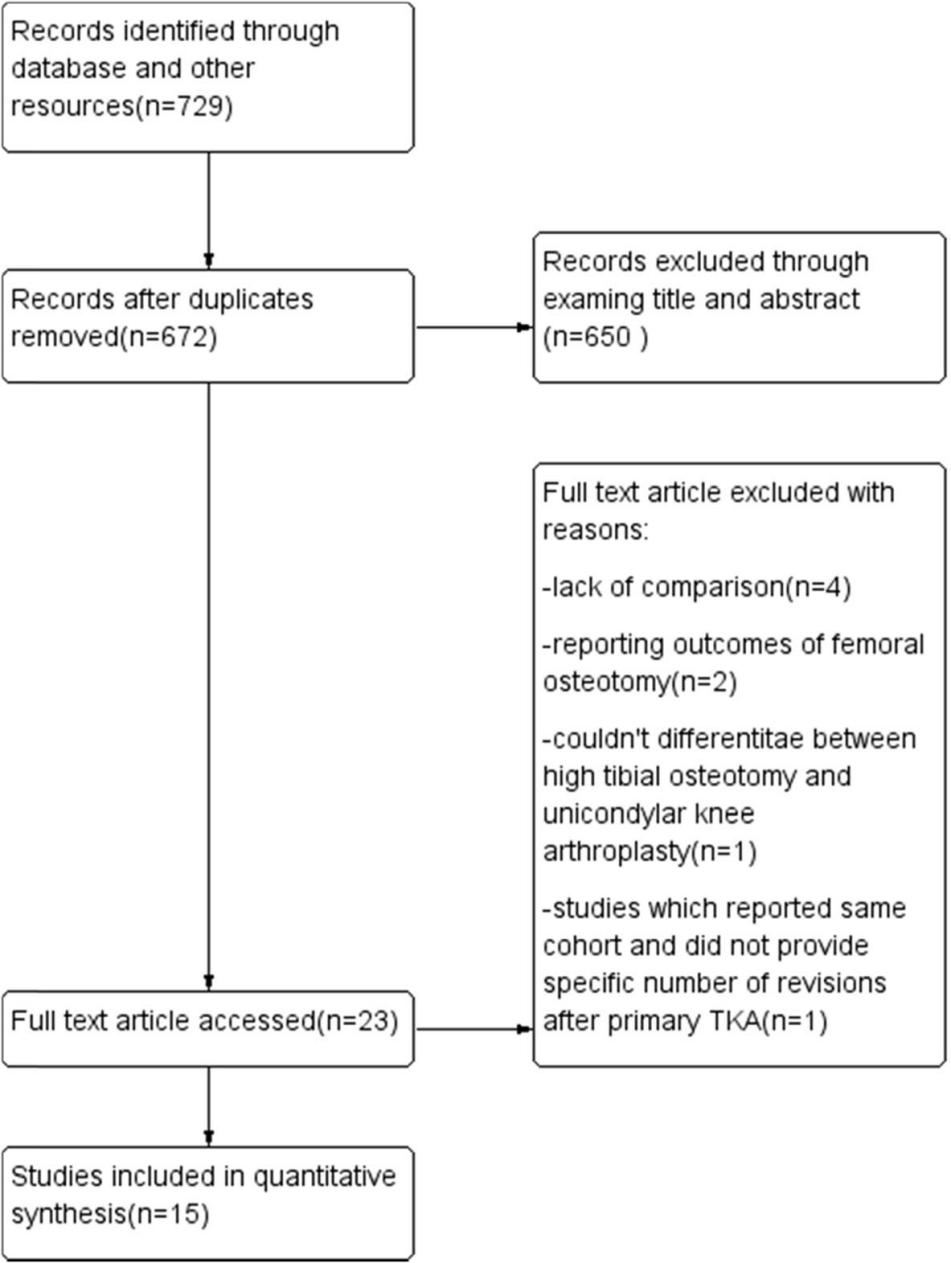
Flow diagram

A total of 15 studies were included in our analysis after a comprehensive search and screening (Fig. 1), the basic characteristics of which are listed in Table 1. All studies compared the clinical outcomes between TKA following HTO (HTO-TKA) and primary TKA. Quality assessment was conducted by two independent researchers according to the NOS quality score. All included studies yielded moderate quality, with an average score of 6 (ranging from 5 to 8).

**Table 1:** Study characteristics

Study	Year	Country	HTO-TKA	TKA	NOS scale
Amendola	2010	Italy	29	29	*******
Bergenudd	1997	Sweden	19	111	*****
Efe	2010	Germany	41	41	********
Erak	2011	Canada	34	1315	******
Haddad	2000	UK	50	50	******
Haslam	2007	UK	51	51	******
Karabatsos	2002	Canada	22	21	*******
Kazakos	2008	Greece	38	38	*******
Meding	2011	USA	39	39	******
Van	2007	Netherland	14	14	******
Pearse	2012	New Zealand	711	34,369	*****
DAHL	2016	Sweden	119	5013	*****
Badawy	2015	Bergen	1399	31,077	*****
Niinimaki	2014	Finland	1036	4143	******
El-Galaly	2018	Denmark	1044	63,763	*****

Most included studies had a minimum follow-up of 1 years (ranging from 1 to 14 years) except 3 registry based studies which did not specify follow-up time [5, 7, 9]; 4646 cases of TKA following HTO and 140,074 cases of primary TKA were included in our study. Parameters, including revision, complication, radiographic outcome and functional outcome were analysed. It was found in our initial screening process that two studies reported on the same cohort; requisite information was gathered and the study with quantitative data of revision cases after primary TKA and with the latest follow-up was included [10, 14]. Four studies [5, 7–9], which included 3391 cases of TKA following HTO and 133,352 cases of primary TKA, were registry-based studies that reported the survival and complications of the 2 groups. In 5 studies [6, 10, 15–17], all cases from the HTO-TKA group underwent lateral closing wedge osteotomy, whereas 1 study [11] reported 42 lateral closing wedge osteotomies and 8 dome osteotomies performed. Two studies [18, 19] included only cases with opening wedge osteotomy, and the remaining 7 studies [5, 7–9, 20, 21] did not specify which technique was applied.

### Revision

**Fig. 2:**
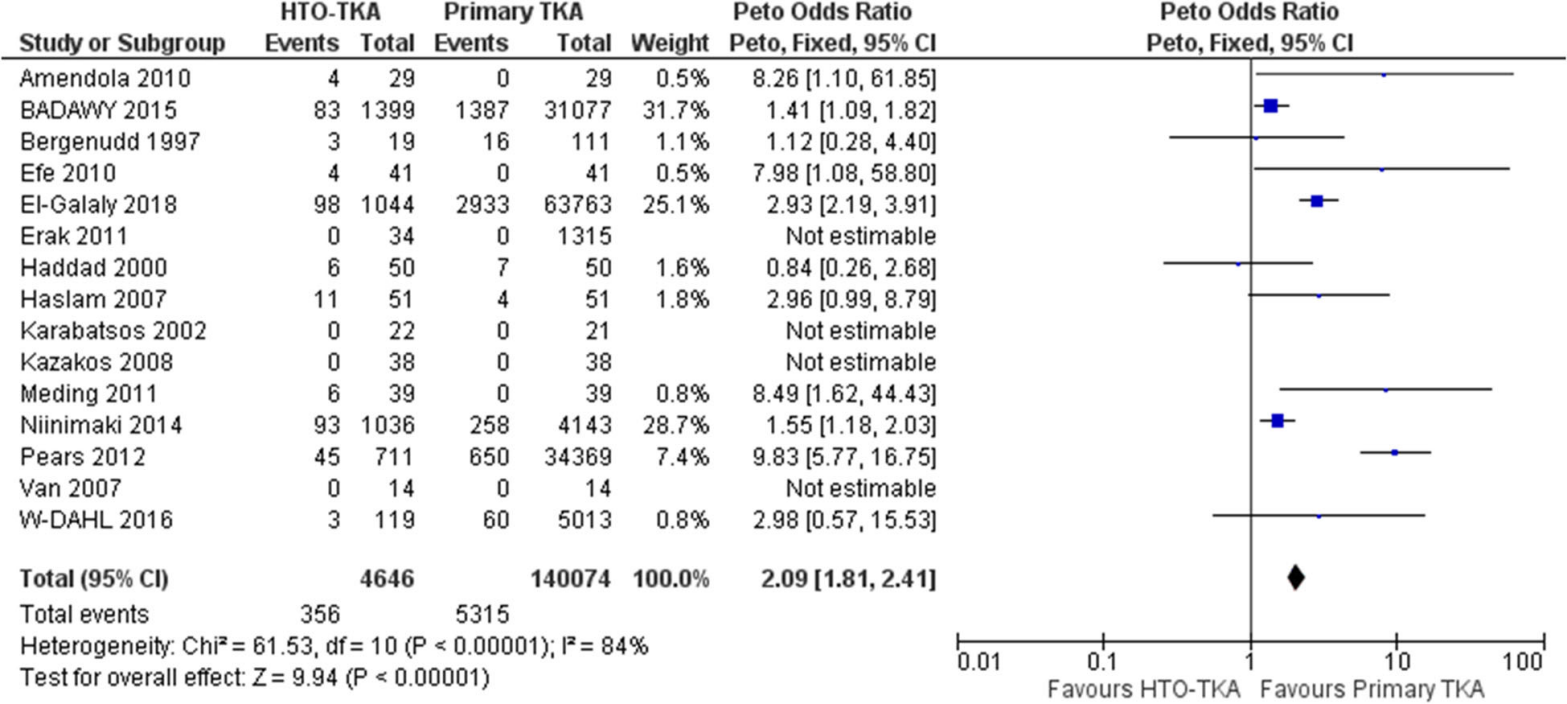
Revision in total

Revision was defined as removal, exchange, insertion of any component or any changes made to an existing component in an existing arthroplasty. After extracting data from all 14 studies, it was found that 356 of 4646(7.66%) cases from the HTO-TKA group and 5315 of 140,074(3.79%) cases from the primary TKA group were revised. Pooled analysis showed significantly more revisions in the HTO-TKA group when comparing with primary TKA (OR 2.09, 95% CI: 1.81–2.41, *P* < 0.0001, I^2^ = 84%) (Fig. 2). The difference is still statistically significant when only cases underwent lateral closing wedge osteotomy were included (OR 4.07, 95% CI: 1.64–10.11, *P* < 0.003, I^2^ = 8%) [6, 10, 15, 16]. The average interval between HTO and subsequent TKA ranges from 4.7 to 8.7 years (Table 2). The average follow-up period ranged from 1 to 8 years, except for 3 registry based studies [5, 7, 9] which did not specify follow-up time (Table 2). Seven included studies reported revision rate between 9 to 16%, 4 studies reported no cases of revision in HTO-TKA group, 1 study reported 21.6% of the HTO-TKA cases underwent revision, 3 studies reported revision rate between 2.5 to 5.9% (Table 2). Eight studies reported reasons for revision in both groups, aseptic loosening (16.78% in HTO-TKA, 22.39% in primary TKA) and deep infection (22.70% in HTO-TKA, 26.54% in primary TKA) were major reasons for revision in both groups.7 studies [5, 6, 8–11, 21] reported on infection rate, the infection rate in HTO-TKA group is 1.4% comparing to 1.0% in primary TKA group. Pooled analysis showed significantly higher risk of infection in HTO-TKA group (OR 1.50, 95% CI: 1.06–2.11, *P* = 0.02, I2 = 62%).

**Table 2:** Revisions in HTO-TKA group

Revision in HTO-TKA group								
	Amendola	BADAWY	Bergenudd	Efe	El-Galaly	Erak	Haddad	Haslam
Revision Cases	4/29	83/1399	3/19	4/41	98/1044	0/34	6/50	11/51
Revision Rate	13.8%	5.9%	15.8%	9.8%	9.4%	0	12%	21.6%
Average Follow-up yrs.	8 (3–13)	NS	6 (4–9)	7 (4–10)	NS	3.4 (2–8)	6.2 (5–10)	> 5
Average interval yrs.	8.39	NS	NS	7.16	NS	4.7	7.3	4.8
	Karabatsos	Kazakos	Meding	Niinimaki	Pears	W-DAHL	Van	
Revision Cases	0/22	0/38	6/39	93/1036	45/711	3/119	0/14	
Revision Rate	0	0	15.4%	9.0%	6.3%	2.5%	0	
Average Follow-up yrs.	5.2	4.5 (3–8)	14	> 1	NS	> 3	> 2	
Average interval yrs.	8.4	7.3	8.7	NS	NS	NS	NS	

### Surgical complexity and solution

Some studies reported prolonged surgical time in the HTO-TKA group; pooled analysis was unattainable because most studies reported only the average surgical time without standard deviation [5, 9, 11, 16, 18, 21]. Perioperative blood loss was also higher, as reported in 2 studies [15, 21]. Seven of the included studies [7, 9, 11, 15, 17–19] reported solutions to surgical complexity in the HTO-TKA group; these are listed in Table 3. Three hundred thirty-four of 1422 (23.5%) cases required stemmed implant. Difficulty during exposure and balancing were also noted in some studies [5, 11, 15, 19].

**Table 3:** Surgical complexity during conversion to TKA (extra measures required)

	Component		Exposure		Balancing			Synovectomies
	Stemed implant	Wedge	Rectus snip	Unattainable patella eversion	Lateral release	Medial release	Medial tightening	
Required	334	4	13	11	31	3	1	4
Total	1422	1422	122	54	125	34	41	41
Percentage	23.49%	0.28%	10.66%	20.37%	24.80%	8.82%	2.44%	9.76%

### Radiographic outcome

Several studies [6, 18, 20] compared radiographic outcome between the 2 groups. The HTO-TKA group showed significantly more tibial component loosening and impingement than the primary TKA group did. No differences were found between groups in terms of femoral component (α), tibial component (β), femoral component flexion (γ) and loosening of the femoral component (Table 4).

**Table 4:** Radiographic outcome

	OR	95%CI	*P*	I^2^
Loosening of femoral component	2.15	0.98,4.71	0.05	0%
Loosening of tibial component	3.14	1.33,7.43	0.009	75%
Impingement	11.97	3.46,41.43	< 0.001	0%

*Impingement* Impingement of tibial stem on the lateral tibial cortex

### Publication bias

**Fig. 3:**
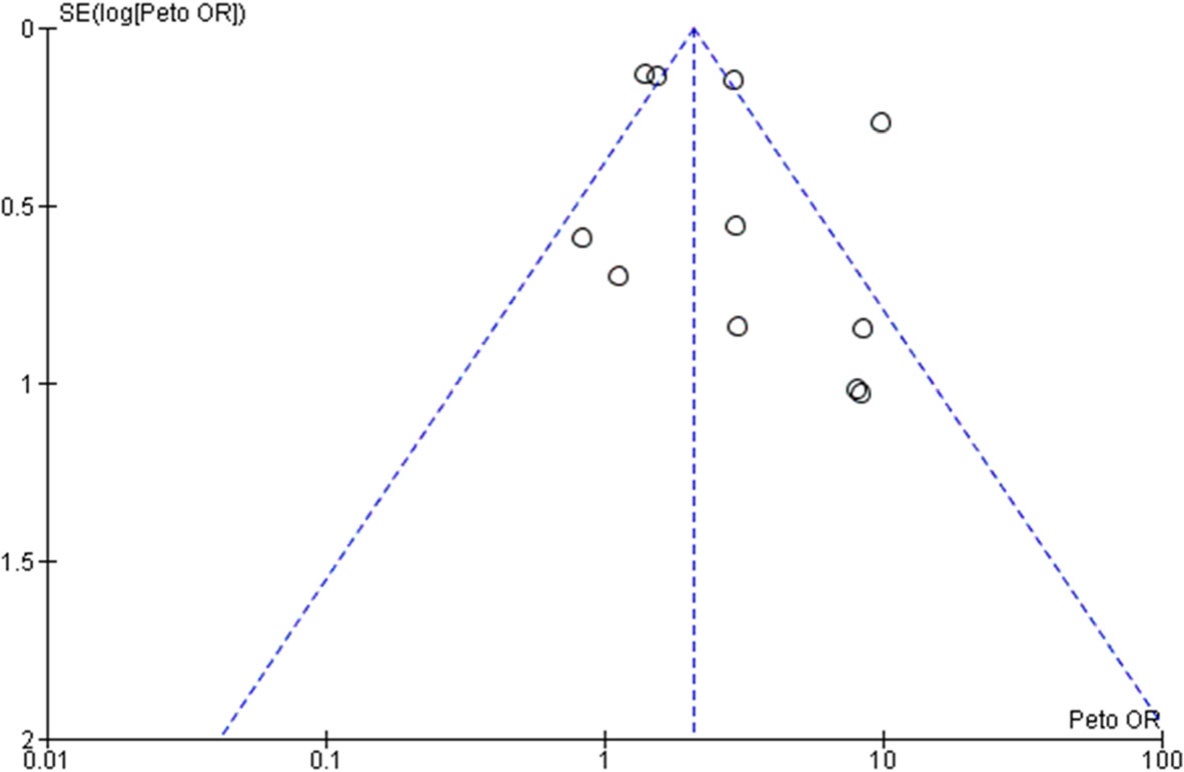
Funnel plot

**Fig. 4:**
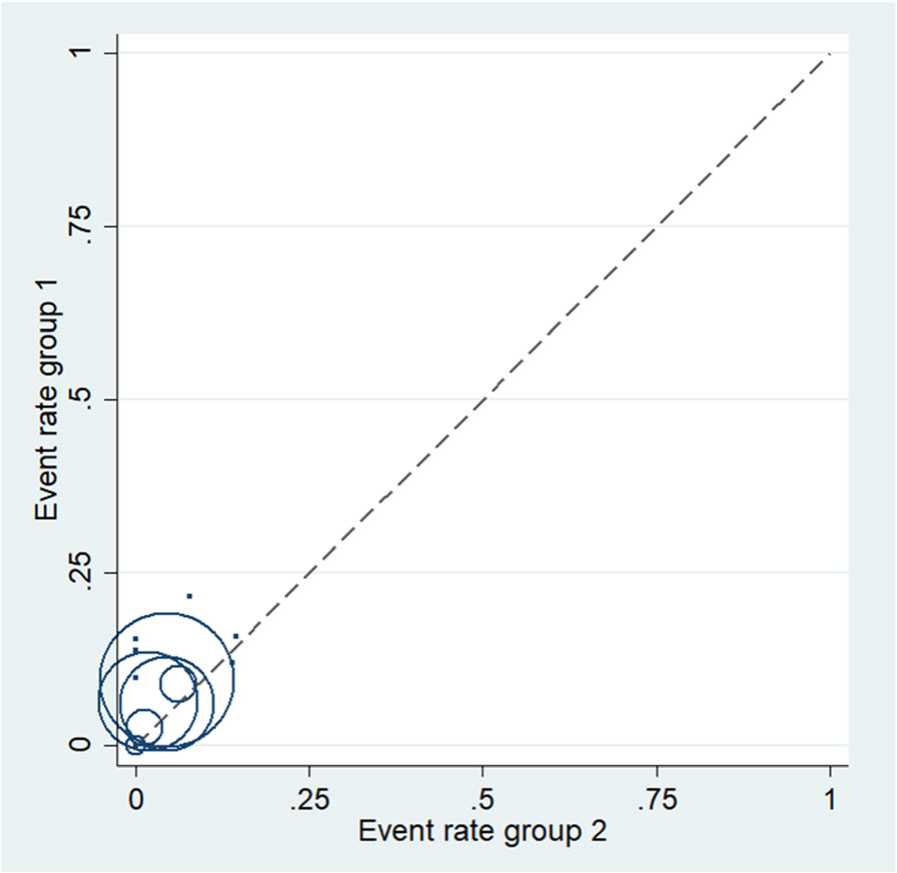
L’abbe plot

Funnel plot (Fig. 3) was conducted in the analysis of total revision, which showed that most studies were within 95% CIs, leaving 2 studies on the edge and 2 studies outside the edge. Slight asymmetry was also noted in the funnel plot. L’abbe test (Fig. 4) was then conducted to assess heterogeneity among different studies in terms of local recurrence, which showed low heterogeneity among included studies.

### Sensitivity analysis

Sensitivity analysis was conducted by excluding studies with fewer than 50 cases included [16, 17] which did not show a significant impact on the results.

## Discussion

High tibial osteotomy was introduced in 1969 by Jackson and Waugh and has become a well-established procedure for unicompartmental knee osteoarthritis since then. The biomechanical rationale for this procedure is correction of malalignment and redistribution of stress on the joint [22].

The classic indication for high tibial osteotomy includes unicompartmental osteoarthritis of the knee and is especially recommended for young patients with high activity demands [23, 24]. For properly selected patients, studies have proven that it offers satisfactory pain relief and functional outcome. However, clinical improvement wears out over time and the majority of patients who underwent this procedure were relatively young. Previous researches have reported subsequent TKAs were required in up to 30% of these cases [25]. Concerns were raised that whether these HTO-TKA would provide comparable survival comparing to primary TKA. There have been conflicting reports regarding this issue. A previous meta-analysis, consisting of 11 studies with 421 HTO-TKAs and 1749 primary TKAs found no significant differences in terms of revision, complications and functional outcome [12]. In our analysis, 15 studies with 4646 HTO-TKAs and 140,074 primary TKAs were included, the substantial increase of sample size may help to investigate low-incidence event such as revision.

Pooled-analysis showed significantly more revisions and complications in the HTO-TKA group, which may be due to following factors: 1.In our analysis, aseptic loosening was the leading cause for revision in HTO-TKA group. Robertsson [26] et al. reported more stemmed implants were required during the conversion from HTO to TKA. Stemmed implant is recommended in these cases because its ability to offer extra rotational stability and avoid stress shielding. Two studies [11, 15] included in our analysis reported impingement between tibial stem and lateral tibial cortex, although it was stated that it appeared to not contribute to early failure. 2.Intraoperative exposure in cases with previous HTO can be more difficult than those of primary TKA. Nizard et al. [27] reported scar tissue between the patellar tendon and the proximal anterior tibia, which made the eversion of patella difficult. Measures including lateral release, rectus snip were applied in included studies. Still, unattainable patella was reported [16, 19] and inadequate exposure may lead to inaccuracy in many aspects during surgery. 3.Malalignment is another common complications encountered in HTO-TKAs, especially in overcorrection after varus tibial osteotomy according to Meding et al. [10]. The joint line on the tibial side become valgus and the bone deficiency on the tibial side can be confusing. The use of traditional method to determine femoral component rotation is often misleading in these cases and internal rotation of the femoral component is suggested.4.Kazakos et al. [15] found more Patella baja in the HTO-TKA group, which could lead to anterior knee pain and eventually revision. This might be due to patella tendon contracture and that the distance between joint line to tibial tuberosity decreased after osteotomy. In our series, based on available data, 10 out of the 304 revised TKA after HTO were patella-related. Patella arthroplasty might be a way to prevent future anterior knee pain and patella-related revision. 5.Persistent pain is more prominent in the HTO-TKA group. Patella baja, excessive soft-tissue release and malalignment may be contributing factors [20]. Impingement between the tibial stem and the tibial cortex might cause pain as well. 6.HTO-TKA group was also subjected to increased surgical time [11], hence to increased risk of infection. In our analysis, the infection rate in HTO-TKA group is 1.4% comparing to 1.0% in primary TKA group, pooled analysis showed significantly higher risk of infection in the HTO-TKA group. In general, the prior osteotomy complicated the anatomical structure of the knee, resulting in varying degrees of deformity, remaining hardware, bone loss and soft-tissue imbalance, which require extra caution and different techniques comparing to primary TKA.

Different osteotomy techniques might influence the risk for subsequent TKA; our analysis showed that prior lateral closing wedge osteotomy also led to significantly more revisions. Comparing to opening wedge HTO, it has been reported in the conversion to TKA from closing wedge HTO, the mechanical axis might be laterally displaced and the tibial insert is more likely to impinge on endosteal cortex, hence a tibial insert with smaller stem is recommended especially in closing wedge HTO [28]. However with the development of computer assisted tibial osteotomy, some traditional difficulties, such as achieving accurate alignment and preventing unintended changes in tibial slope encountered during osteotomy can be solved with computer assisted planning and navigation [29]. Patients underwent computer assisted tibial osteotomy may not present the same surgical challenges as traditional osteotomy and more researches are needed in this field.

Amendola et al. [20] argued that the time in which the subsequent TKA was performed was also crucial and that surgeons tend to have a better understanding of the technical difficulties to achieve comparable results over time. In our series, four studies were published within the last 5 years; three [5, 9] of them suggested similar survival between 2 groups, whereas one study [8] reported survival in favour of primary TKA. Pooled analysis from these 4 studies still showed significantly more revision in the HTO-TKA group.

Several methods, including lateral release of the lateral alar ligament of the patella; quadriceps snips; even osteotomy of the anterior tibial tuberosity were suggested in order to tackle the technical difficulties encountered during the conversion to TKA due to the presence of scarring tissue which poses substantial challenge during exposure [30–33]. In our series, rectus snips remain the most common technique to achieve satisfactory exposure. Amendola et al. [20]. verified that in cases with prior osteotomy, the medial plateau is higher than the lateral plateau in anteroposterior (AP) radiographs. Erak et al. [20]. reported preoperative patella baja relating to difficulty with patella eversion. In order to balance the knee, release of lateral ligament was also suggested due to the extra medial dissection to remove osteotomy hardware [20]. The bone resected in the lateral region must be minimal to avoid a large defect. Wedges were used in 4 out of 711 cases as reported by Pearse et al. [7]. A high percentage of stemmed implants were used in the HTO-TKA group; this may result from the need to avoid a potential stress riser. Ligament balancing is crucial in cases with prior osteotomy; the fibrosis and loss of soft tissue may lead to instability [33]. The most common balancing technique in our analysis was lateral release (31/125). Difficulty during exposure, resection and component positioning contributed to the prolonged surgical time and led to increased blood loss.

Few studies reported on radiographic outcome between the two groups. Based on available data, more loosening of the tibial component and impingement were noted in the HTO-TKA group, which correlates with our analysis of revision. No significant differences were found in terms of alignment. Kazakos et al. [15] reported 16 cases of patella baja in the HTO-TKA group, with only two in the control group. Other studies also stated patella baja to be more common after HTO, but they did not find any relevance between patella baja and the clinical outcome of subsequent TKA [32, 34].

Our study has several limitations. (1) All included studies were retrospective studies and registry based studies whereas no RCT was included, which limited the quality of this meta-analysis. (2) The mean follow-up was not consistent among studies; revisions might not be required until 10–20 years later. (3) Some studies only reported mean and range for parameters such as surgical time, blood loss and functional score, and we were unable to conduct pooled analysis based on these data. (5) Some causes of revision were marked as unknown in some studies, which influenced our analysis of the causes of revision.

The strengths of this meta-analysis include the following. (1) This study focused on the risk of revisions and further investigate surgical complexity and radiographic outcomes with more cases involved and explored potential causes for the differences between groups. Most studies reported comparable survival outcome between the two groups, whereas pooled analysis of gathered data revealed statistically significant results. (2) Due to the lack of relevant studies and cohort, this meta-analysis gathers valuable information to conduct a quantitative analysis and yielded result inconsistent with the first meta-analysis on this subject. (3) Randomised trials were not feasible considering our research purpose; this study gathers data from retrospective studies and provides the best evidence available. (4) Relevant articles were screened carefully by two independent researchers, using a wide range of search terms. (5) Previous meta-analysis on this subject included 421 HTO-TKAs and 1749 primary TKAs, while our study included 4646 HTO-TKAs and 140,074 primary TKAs, the increased sample size allow us to assess small-incidence event such as revision with more accuracy. (6) Considering the wide variation of publishing time of included studies, pooled analysis of studies published within the last five 5 years yielded consistent finding. (7) Clear inclusion and exclusion criteria were utilized.

## Conclusion

High tibial osteotomy offers satisfactory pain relief and functional outcome in selected patients with high activity demand. However, the need for subsequent TKA should be noted, which might be a technically challenging procedure with significantly higher risk of revision comparing to primary TKA.

## Data Availability

Not applicable. The data used for analysis was retrieved from openly published studies listed in our manuscript.

## Abbreviations

HTO: High tibial osteotomy
TKA: Total knee arthroplasty

## References

[1] Seil R, van Heerwaarden R, Lobenhoffer P, Kohn D (2013). The rapid evolution of knee osteotomies. Knee Surg Sports Traumatol Arthrosc.

[2] Gstottner M, Pedross F, Liebensteiner M, Bach C (2008). Long-term outcome after high tibial osteotomy. Arch Orthop Trauma Surg.

[3] Hui C, Salmon LJ, Kok A, Williams HA, Hockers N, van der Tempel WM, Chana R, Pinczewski LA (2011). Long-term survival of high tibial osteotomy for medial compartment osteoarthritis of the knee. Am J Sports Med.

[4] Spahn G, Hofmann GO, von Engelhardt LV, Li M, Neubauer H, Klinger HM (2013). The impact of a high tibial valgus osteotomy and unicondylar medial arthroplasty on the treatment for knee osteoarthritis: a meta-analysis. Knee Surg Sports Traumatol Arthrosc.

[5] El-Galaly A, Nielsen PT, Jensen SL, Kappel A (2018). Prior high tibial osteotomy does not affect the survival of total knee arthroplasties: results from the Danish knee arthroplasty registry. J Arthroplast.

[6] Haslam P, Armstrong M, Geutjens G, Wilton TJ (2007). Total knee arthroplasty after failed high tibial osteotomy long-term follow-up of matched groups. J Arthroplast.

[7] Pearse AJ, Hooper GJ, Rothwell AG, Frampton C (2012). Osteotomy and unicompartmental knee arthroplasty converted to total knee arthroplasty: data from the New Zealand joint registry. J Arthroplast.

[8] Niinimaki T, Eskelinen A, Ohtonen P, Puhto AP, Mann BS, Leppilahti J (2014). Total knee arthroplasty after high tibial osteotomy: a registry-based case-control study of 1,036 knees. Arch Orthop Trauma Surg.

[9] Badawy M, Fenstad AM, Indrekvam K, Havelin LI, Furnes O (2015). The risk of revision in total knee arthroplasty is not affected by previous high tibial osteotomy. Acta Orthop.

[10] Meding JB, Wing JT, Ritter MA (2011). Does high tibial osteotomy affect the success or survival of a total knee replacement?. Clin Orthop Relat Res.

[11] Haddad FS, Bentley G (2000). Total knee arthroplasty after high tibial osteotomy: a medium-term review. J Arthroplast.

[12] Ramappa M, Anand S, Jennings A (2013). Total knee replacement following high tibial osteotomy versus total knee replacement without high tibial osteotomy: a systematic review and meta analysis. Arch Orthop Trauma Surg.

[13] Gaillard R, Lording T, Lustig S, Servien E, Neyret P (2017). Total knee arthroplasty after varus distal femoral osteotomy vs native knee: similar results in a case control study. Knee Surg Sports Traumatol Arthrosc.

[14] Meding JB, Keating EM, Ritter MA, Faris PM (2000). Total knee arthroplasty after high tibial osteotomy. Clin Orthop Relat Res.

[15] Kazakos KJ, Chatzipapas C, Verettas D, Galanis V, Xarchas KC, Psillakis I (2008). Mid-term results of total knee arthroplasty after high tibial osteotomy. Arch Orthop Trauma Surg.

[16] Karabatsos B, Mahomed NN, Maistrelli GL (2002). Functional outcome of total knee arthroplasty after high tibial osteotomy. Can J Surg.

[17] van Raaij TM, Bakker W, Reijman M, Verhaar JA (2007). The effect of high tibial osteotomy on the results of total knee arthroplasty: a matched case control study. BMC Musculoskelet Disord.

[18] Efe T, Heyse TJ, Boese C, Timmesfeld N, Fuchs-Winkelmann S, Schmitt J, Theisen C, Schofer MD (2010). TKA following high tibial osteotomy versus primary TKA--a matched pair analysis. BMC Musculoskelet Disord.

[19] Erak S, Naudie D, MacDonald SJ, McCalden RW, Rorabeck CH, Bourne RB (2011). Total knee arthroplasty following medial opening wedge tibial osteotomy: technical issues early clinical radiological results. Knee.

[20] Amendola L, Fosco M, Cenni E, Tigani D (2010). Knee joint arthroplasty after tibial osteotomy. Int Orthop.

[21] Bergenudd H, Sahlstrom A, Sanzen L (1997). Total knee arthroplasty after failed proximal tibial valgus osteotomy. J Arthroplast.

[22] Jackson JP, Waugh W, Green JP (1969). High tibial osteotomy for osteoarthritis of the knee. J Bone Joint Surg Br.

[23] Mancuso F, Hamilton TW, Kumar V, Murray DW, Pandit H (2016). Clinical outcome after UKA and HTO in ACL deficiency: a systematic review. Knee Surg Sports Traumatol Arthrosc.

[24] Dettoni F, Bonasia DE, Castoldi F, Bruzzone M, Blonna D, Rossi R (2010). High tibial osteotomy versus unicompartmental knee arthroplasty for medial compartment arthrosis of the knee: a review of the literature. Iowa Orthop J.

[25] Schuster P, Gesslein M, Schlumberger M, Mayer P, Mayr R, Oremek D, Frank S, Schulz-Jahrsdorfer M, Richter J (2018). Ten-year results of medial open-wedge high tibial osteotomy and chondral resurfacing in severe medial osteoarthritis and varus malalignment. Am J Sports Med.

[26] Robertsson O, W-Dahl A (2015). The risk of revision after TKA is affected by previous HTO or UKA. Clin Orthop Relat Res.

[27] Nizard RS, Cardinne L, Bizot P, Witvoet J (1998). Total knee replacement after failed tibial osteotomy: results of a matched-pair study. J Arthroplast.

[28] Kuwashima U, Tashiro Y, Okazaki K, Mizu-Uchi H, Hamai S, Murakami K, Iwamoto Y (2017). Comparison of the impact of closing wedge versus opening wedge high tibial osteotomy on proximal tibial deformity and subsequent revision to total knee arthroplasty. Knee Surg Sports Traumatol Arthrosc.

[29] Song SJ, Bae DK (2016). Computer-assisted navigation in high tibial osteotomy. Clin Orthop Surg.

[30] Whiteside LA (1995). Exposure in difficult total knee arthroplasty using tibial tubercle osteotomy. Clin Orthop Relat Res.

[31] Garvin KL, Scuderi G, Insall JN (1995). Evolution of the quadriceps snip. Clin Orthop Relat Res.

[32] Windsor RE, Insall JN, Vince KG (1988). Technical considerations of total knee arthroplasty after proximal tibial osteotomy. J Bone Joint Surg Am.

[33] Katz MM, Hungerford DS, Krackow KA, Lennox DW (1987). Results of total knee arthroplasty after failed proximal tibial osteotomy for osteoarthritis. J Bone Joint Surg Am.

[34] Mont MA, Alexander N, Krackow KA, Hungerford DS (1994). Total knee arthroplasty after failed high tibial osteotomy. Orthop Clin North Am.

